# Maternal deprivation induces alterations in cognitive and cortical function in adulthood

**DOI:** 10.1038/s41398-018-0119-5

**Published:** 2018-03-27

**Authors:** Sarine S. Janetsian-Fritz, Nicholas M. Timme, Maureen M. Timm, Aqilah M. McCane, Anthony J. Baucum II, Brian F. O’Donnell, Christopher C. Lapish

**Affiliations:** 1 Department of Psychology, Indiana University-Purdue University Indianapolis, Indianapolis, IN USA; 2 Department of Biology, Indiana University-Purdue University Indianapolis, Indianapolis, IN USA; 3 Department of Psychological and Brain Sciences, Indiana University, Bloomington, IN USA; 4 Indiana University School of Medicine Stark Neuroscience Institute, Indianapolis, IN USA; 5 Indiana University-Purdue University Indianapolis School of Science Institute for Mathematical Modeling and Computational Sciences, Indianapolis, IN USA

## Abstract

Early life trauma is a risk factor for a number of neuropsychiatric disorders, including schizophrenia (SZ). The current study assessed how an early life traumatic event, maternal deprivation (MD), alters cognition and brain function in rodents. Rats were maternally deprived in the early postnatal period and then recognition memory (RM) was tested in adulthood using the novel object recognition task. The expression of catechol-o-methyl transferase (COMT) and glutamic acid decarboxylase (GAD67) were quantified in the medial prefrontal cortex (mPFC), ventral striatum, and temporal cortex (TC). In addition, depth EEG recordings were obtained from the mPFC, vertex, and TC during a paired-click paradigm to assess the effects of MD on sensory gating. MD animals exhibited impaired RM, lower expression of COMT in the mPFC and TC, and lower expression of GAD67 in the TC. Increased bioelectric noise was observed at each recording site of MD animals. MD animals also exhibited altered information theoretic measures of stimulus encoding. These data indicate that a neurodevelopmental perturbation yields persistent alterations in cognition and brain function, and are consistent with human studies that identified relationships between allelic differences in COMT and GAD67 and bioelectric noise. These changes evoked by MD also lead to alterations in shared information between cognitive and primary sensory processing areas, which provides insight into how early life trauma confers a risk for neurodevelopmental disorders, such as SZ, later in life.

## Introduction

The prenatal or early neonatal period is critical for brain development and can be powerfully influenced by environmental factors^1^. Early-life adverse events such as malnutrition, maternal separation, or viral infection^2–5^ may disrupt brain development^6^, possibly leading to psychopathology later in life, including schizophrenia (SZ)^7,8^. Therefore, determining how early life trauma alters neural circuit composition/function is important to understand the link with an increased risk for psychopathology.

An often-used animal model of early life trauma is maternal deprivation (MD), which is based on exposure to stress in early postnatal life (24-h deprivation on postnatal day (PD) 9)^9^. Early-life trauma on PND 9 can induce neurochemical and molecular changes. More specifically, following MD, functioning of the hypothalamic-pituitary-adrenal axis is blunted^10^, which can be a result of increased 5-HT in the hippocampus (HC), striatum, and prefrontal cortex (PFC)^8^. Altered hippocampal NMDA receptor subunit expression has also been observed following MD^11^, which is associated with impairments in learning and memory later in life^12^. Interestingly, administering a NMDA receptor antagonist (Memantine), prevents impaired social cognition typically observed in adulthood following MD^13^, further supporting that early life trauma can alter the glutamatergic system. Lastly, decreases in brain derived neurotrophic factor (BDNF) have been detected in the HC, which is important for brain maturation including neuronal survival, plasticity, and differentiation^14,15^. Collectively, these data indicate that MD has broad effects on neurotransmitter systems, which is likely key for the expression of altered cognitive function later in life.

Several short-term and long-term behavioral disturbances are observed following MD, including an increased depressive-like phenotype^16,17^ and changes in locomotor activity^16,18^. “Psychomotor agitation” is a common pathology observed in SZ^19^, and has been widely studied as hyperlocomotion in animal models of the disease (for review see 20). Although scarcely explored, studies also report impairments in recognition memory (RM)^21,22^, while others find no differences^17,23^. RM is among the most severe cognitive impairments observed in patients with SZ^24,25^. In the current study, Novel Object Recognition (NOR) was used as a validated and translational measure of RM^26^.

Basic forms of information processing are also impaired following MD, including sensorimotor gating measured by prepulse and latent inhibition^27–29^, which are thought to contribute to cognitive impairments^30^. Given that patients with SZ have deficits in information processing^31^, it has been argued that the most effective animal models of SZ are those that model deficits in sensorimotor gating^32^. Understanding how the flow of information between brain regions might be altered following MD is of paramount importance for understanding how SZ affects normal brain functions. Towards this goal, information theoretic approaches were used in the current study to assess neural encoding within and across the cortex.

Increases in the variability of neural signals measured via electroencephalogram (EEG) (referred to herein as bioelectric noise) is observed in SZ and has been argued to reflect an endophenotypic marker of SZ^33^. A negative correlation is observed between bioelectric noise in the mPFC and working memory^33,34^. This noise endophenotype is also associated with variations of the Val^158^/Met^158^ allele of the catechol-o-methyl transferase (COMT) gene^33^, which is involved in regulating catecholamines^35,36^. Clearly identifying an association between the Val^158^/Met^158^ allele and SZ has been difficult though, with some groups reporting associations^37,38^ while others failed to find one^39^. However, several studies observed that this variant modulates a wide range of cognitive and neurophysiological phenotypes in humans^40,41^ but see^42^. Moreover, a longitudinal study found that maternal stress increased risk for behavioral disorders in offspring that were Met^158^ homozygotes^43^. In contrast to the extensive literature on these genetic effects, the role of developmental factors on subsequent expression of COMT has been infrequently studied.

Alterations in the gamma-aminobutyric acid (GABA) system are also observed in SZ^44^. Specifically, patients exhibit decreased expression of glutamic acid decarboxylase 67 (GAD67), an enzyme involved in the decarboxylation of glutamate to GABA^45^. Val^158^ homozygotes also have genetic variations in GAD67^46^. Epistasis between COMT and GAD67 is observed^47^, which is interesting given the relationship between DA and GABA in tuning of cortical circuits^48^. However, no study to date has assessed the effects of MD on COMT and GAD67, concurrently.

The current study examined a number of behavioral, biochemical, and neurophysiological markers to determine how MD alters brain structure and function in adulthood.

## Materials and methods

### Subjects

**Fig. 1:**
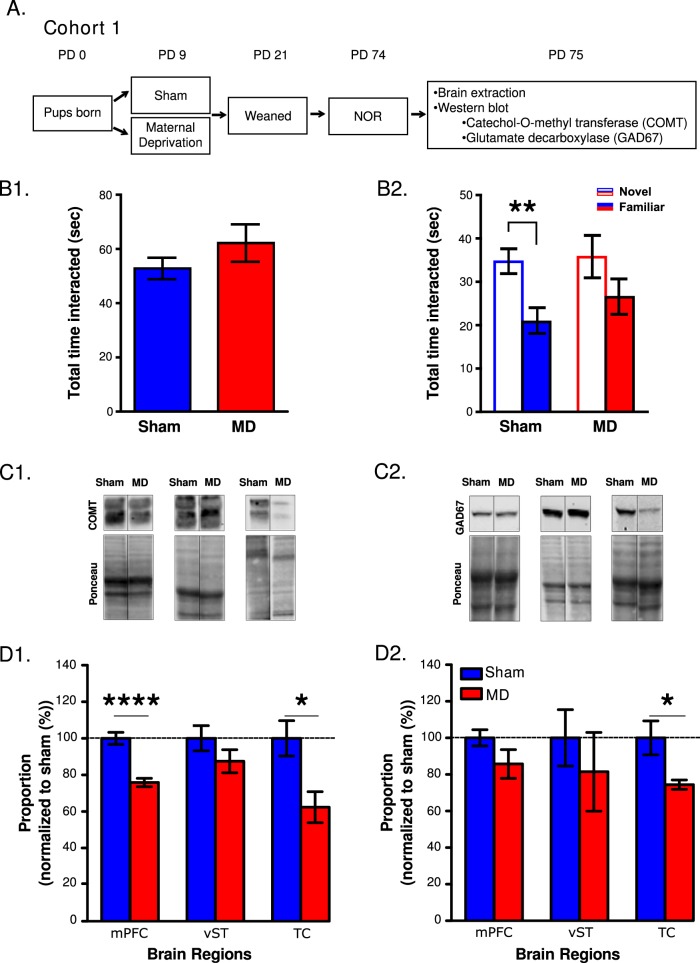
Experimental timeline, novel object recognition (NOR), and protein expression following MD or sham. **a** A timeline of experiments from cohort 1. **b1** Total time interacted (s) with both objects collectively and **b2** with the novel or familiar object, separately (Bonferroni corrected planned comparison, **p < 0.01; significantly different than familiar object). **c1** Examples of Catechol-o-methyl transferase (COMT) and **c2** glutamic acid decarboxylase (GAD67) western blot, and associated Ponceau stain from the medial prefrontal cortex (mPFC), ventral striatum (vST), and temporal cortex (TC) from a sham and MD animal. **d1** COMT and **d2** GAD67 protein expression (bar graphs) from the mPFC, vST, and TC between groups. Protein expression is normalized to shams (dashed line). (Unpaired two-tailed *t*-tests, *p < 0.05, ****p < 0.0001; significantly different than shams). All data are depicted as mean ± SEM

**Fig. 2:**
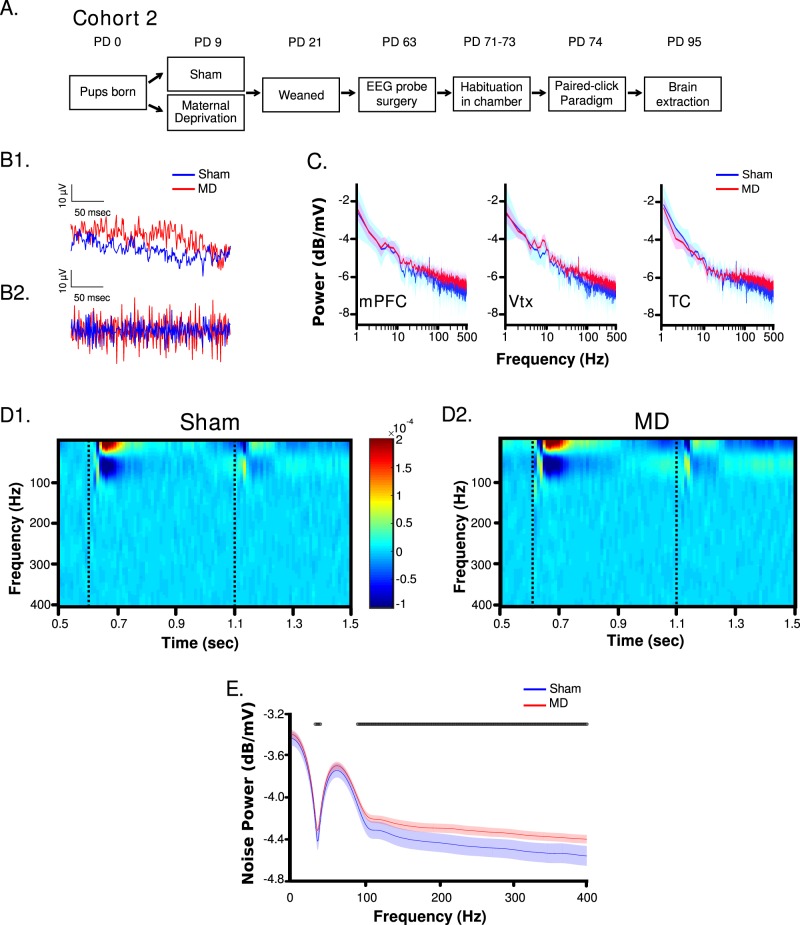
Experimental timeline, power and noise power between MD and sham. **a** A timeline of experiments from cohort 2. **b1** Representative raw voltage traces from a sham and MD animal. **b2** The change in voltage between adjacent time points from b1. **c** The mean power spectra (solid line) ± SEM (shaded portion) of MD (red) and sham (blue) animals from the mPFC, vertex, and TC dEEG’s. **d1** Mean mPFC spectrogram from sham and **d2** MD animals (dotted lines = onset of clicks). **e** Average Noise Power ± SD (shaded region) in the mPFC for each frequency band. Dots denote regions of significant differences (FDR corrected unpaired *t*-tests t’s(22) < 1.63, p’s < 0.01)

Timed pregnant Sprague-Dawley rats (*n* = 17) (Harlan, Indianapolis, IN) arrived approximately one-week prior to giving birth and were individually housed and maintained on a reverse light/dark schedule with ad libitum access to food/water. On PD0 when pups were born, litters were culled to 8 pups (4 females and 4 males when possible) and each litter was placed in a separate cage. On PD21, males from the same litter were group housed to 2–3 and females were euthanized using CO_2_. Rats were individually housed on PD60. For cohort 1, animals (MD *n* = *13*; sham *n* = *10*) were weighed once a week until PD71 (brains were extracted on PD75). RM and protein expression were measured in cohort 1 (see Fig. 1a). Animals in cohort 2 (MD *n* = *24*; sham *n* = *12*) were weighed ~2 weeks prior (PD75-85) to brain extractions. Offspring were handled five days a week starting on PD25 until PD72. Locomotor activity, electrophysiological recordings, and information theoretic analyses were assessed in cohort 2 (see Fig. 2a). All procedures were performed and approved in accordance with the Purdue School of Science Animal Care and Use committee’s regulations and conformed to the Guidelines for the Care and Use of Mammals in Neuroscience and Behavioral Research^49^.

### Maternal deprivation

MD procedures were conducted identical to those used in previous experiments^21,27,28,50^. To induce MD, the mother of the litter was removed at 10:00 am on PD9, weighed, and placed in a different cage and room with food and water. Then, each pup was weighed and placed back in the same home cage for 24 h without their mother, food or water. During this period, a heating pad maintained between 30–33 °C was placed under the cage to prevent pups from developing hypothermia. On PD10 at 10:00 am, pups were weighed, the mother was weighed, and she was returned to the original cage with her pups. Food and water were replaced in the cage. For shams, the mother of the litter was removed at 10:00 am, weighed, and then temporarily placed in a different cage and room. Pups were weighed and placed back in their home cage, followed by the mother. Rats were left undisturbed with access to food and water until they were weaned on PD21.

### Novel object recognition

On PD74, novel object recognition (NOR) was used to assess RM in rats (5 total litters) and was conducted identically to a previous study^51^. The open field chamber (86.36 × 93.98 × 31.24 cm^3^) contained Velcro strips ~16.5 cm from one another in two opposite corners of the chamber. Objects used included rubber ducks (8.1 × 6.35 cm^2^), Rubik’s cubes (5.8 × 5.8 cm^2^), circular white cups (7.62 × 4.57 cm^2^), plastic cupcakes (5.08 × 8.89 cm^2^), containers (10.16 × 7.62 × 3.81 cm^3^), and toy trucks (8.9 × 3.81 × 5.08 cm^3^). The order of objects were randomized. One observer manually scored interaction.

Rats explored two different sets of identical objects in trials 1–2 with a 45-min inter-trial interval (ITI). In trial 3, after a 60-min ITI, one object from each previous trial was used. After 60-min, in trial 4, RM was assessed as the rat freely explored a novel object and the familiar object from trial 2. All trials lasted 4 min. Although NOR is typically conducted in two trials (a familiarization and a retention trial), these specific procedures were used to expose animals to the familiar object two times (in trial 2 and in trial 3) before exposing the animal to the novel object, making it more likely for the animal to encode the object.

The dependent variable to assess RM was the amount of time (seconds) that an animal spent interacting with each object. Total exploration time with both objects during Trial 4 was assessed using unpaired *t*-tests to compare differences between MD and sham groups. A two-way RM ANOVA was used to assess differences in exploration time with each object between groups. Bonferroni corrected planned comparisons (alpha adjusted to p = 0.025) were conducted to assess differences between time spent exploring each object within each treatment group, separately.

### Western blot

Following cognitive testing, COMT and GAD67 protein expression were assessed in the medial PFC (mPFC) (AP: + 3.2; ML: + 0.6; DV:-2.5) (MD *n* = *8*; sham *n* = *6*), temporal cortex (TC) (AP:-4.5; ML:0.0; DV:-4.0) (MD *n* = *7*; sham *n* = *7*), and ventral striatum (vST) (AP:1.6; ML:1.5; DV:-6.7) (MD *n* = *7*; sham *n* = *7*). Coordinates were chosen based on the rat brain atlas^52^. The mPFC and TC were assessed given their role in cognitive function and sensory gating^53,54^. The vST was assessed as a non-cortical brain region also implemented in the etiology of SZ^55^.

Tissue was placed in Potter-Elvehjem tissue grinder and homogenized in 2% SDS. Protein concentration was determined by using a BCA assay kit (Pierce Biotechnology, Inc., Rockford, IL, USA). Proteins were separated on 12% SDS–Page gels (Bio-Rad) then transferred using a Transblot Turbo System (Bio-Rad Laboratories, USA) to nitrocellulose paper via 1.3A 25 V for 7 min (COMT ~24 Kd) and 15 min for GAD67 (~67 Kd). The blots were then stained with Ponceau S (BP10-10, Thermo Fisher Scientific, Waltham, MA., USA) and scanned as a TIFF image on Epson V700 photo scanner for loading control analysis. For immunostaining, the blot was blocked for 1 h in TBS-T (TBS with 0.1% Tween 20) and 5% nonfat dry milk for 60 min. The blot was then incubated in anti-COMT (BDB611970, BD Transduction Laboratory) and was diluted 1/1000 and anti-GAD (5305, Cell Signaling Technology), which was diluted to 1/100. Then the blot was rinsed in TBS-T with 5% nonfat dry milk. The second antibody (Goat anti-M`ouse Alexa 790 (926–32210, Li-Cor Inc, Lincoln, NE) or Goat anti-Rabbit Alexa 790 (926-32211, Li-Cor Inc, Lincoln, NE) was diluted 1/10,000 in TBS-T with 5% nonfat dry milk. The blot was incubated at room temperature for 1 h on a rocker and then rinsed in TBS. Bands were visualized with the LI-Cor CLx Odyssey Infrared Imaging System (Li-Cor Inc, Lincoln, NE).

To control for potential loading differences, Ponceau signal intensity was assessed between MDs and shams via unpaired two-tailed *t*-tests in each brain region separately^56^. Since each brain region was run on different gels, it was inappropriate to compare groups based on a loading control.

To compare COMT protein levels across groups, COMT was normalized by Ponceau signal intensity and then compared across groups. More specifically, to obtain a normalized value for each animal, the following formula was used: (total COMT/Ponceau signal intensity)/(averaged total COMT/Ponceau signal intensity of all sham rats). Data are presented as “proportion (normalized to sham (%))”. Then, unpaired two-tailed *t*-tests were used to assess differences in protein levels between sham and MD groups in each brain region, separately. Identical data preparation and analyses were conducted for GAD67.

### Surgical procedures

Rats were deprived or left undisturbed and then surgery was performed on PD63. Two-three rats from each litter were randomly selected for surgery (12 total litters). Rats were anesthetized with isoflurane (5% induction, 2% maintenance) and placed into a Kopf stereotaxic frame. The skull was exposed after an incision was made. A depth EEG probe with stainless steel screws were implanted over the mPFC (AP: + 3.2; ML: + 0.6; DV:0.0), vertex (AP:-4.0; ML:+1.0; DV:0.0), and TC (AP:−4.5; ML:0.0; DV:−4.0). Electrical signals were grounded to two skull screws in the interstitial space over the cerebellum. Probes were secured with dental acrylic. Animals had one week to recover from surgery before beginning experiments. At the end of the experiment, animals were euthanized using Urethane via intraperitoneal injection at a dose of 1.5 g/kg dissolved in sterile water in a volume of 0.1 ml/kg. Then, brains were extracted to determine probe placement (PD95).

### Paired-click paradigm

Animals were habituated in chambers (31.75 × 31.75 × 40.64 cm^3^) for 20-min/day (PD 71-73) that were equipped with a white noise (Med Associates; 7.6 × 8.3 cm) and a tone (Mallory Sonalert Products, Inc., Indianapolis, IN; 3.07 × 2.87 cm) generator. On all three habituation days, a tether was connected to the EIB-27 micro to habituate rats to this procedure.

On PD74, dEEG recordings were obtained using a digital Neuralynx Cheetah recording system (Neuralynx; Bozeman, MT). There was a 30 min habituation period where rats were plugged in and freely moved around the chamber with no auditory stimuli present. Then, sensory gating was examined using the paired-click paradigm, with 60 1 ms duration clicks (80 dB, white noise) delivered in pairs with an inter-click interval of 500 ms, and an inter-stimulus interval of 10 s. During electrophysiological recordings, locomotor activity was acquired for 60 min via video camera, recorded in ANY-maze (Wood Dale, IL), and was synchronized with electrophysiological recordings. Mean locomotor activity (cm per second) was measured as a behavioral test to assess whether MD animals exhibited hyperlocomotion compared to shams.

dEEGs were subsampled from 32,556 to 1017 Hz for offline analyses. Each trace was then detrended via mean subtraction. Signals with excessive variance were detected by computing a 99.99% confidence interval for each time step. If any part of the time series fell outside of this bound, the entire trace was excluded from subsequent analyses. To determine mean voltage x time response following each click, −250 ms before and 500 ms after each click was extracted from the recording. The tone-evoked change in voltage was characterized by three peaks and the magnitude of each peak was compared across the first and second click resulting in a ratio of the first to the second response. The gating ratio was defined as:

$$Gating\,ratio = \frac{{\left| {\mathop {\sum }\nolimits_{i = 1}^3 x_{2,i}} \right|/3}}{{\left| {\mathop {\sum }\nolimits_{i = 1}^3 x_{1,i}} \right|/3}}$$

where the numerator refers to the second click and the denominator to the first click. The index *i* refers to the *i’th* component of the triphasic voltage deflection.

For sensory gating analyses, each component of the triphasic peak was extracted from the raw trace by taking the maximum absolute value of the portion of the signal that contained the peak (i.e. 13–16, 25–45, 46–84 ms after tone onset). These values were then assessed via mixed effects ANOVA with treatment as the between subject factor and brain region (mPFC, vertex, TC) and click (1 and 2) as the within subjects factors. Then, multiple comparisons were conducted to assess differences between the first and second click in the different brain regions using Tukey’s post-hoc analyses. To further quantify the gating response, a “gating ratio” was assessed in each brain region and in both treatment groups. This quantity creates a ratio of the overall size of the voltage response to the second click divided by the first, which can be interpreted as a percentage. ANOVA was used to assess differences in gating ratio across all brain regions and between treatment groups, followed by pairwise planned comparisons to detect differences in the gating response in each brain region.

Bioelectric noise was quantified in two ways. First, the average change in voltage ($\left\langle {\Delta V} \right\rangle$) of each electrophysiological recording was calculated as:

$$\Delta V = \frac{1}{n}\mathop {\sum }\limits_{i = 1}^n \sqrt {(v_i - v_{i - 1})^2}$$

where *n* = total number of samples in the voltage trace and *v*_*i*_ = voltage at time *i*. Second, Noise Power^57,58^, was implemented as the change in power (*p*) relative to the mean for each frequency band for a dEEG (*x*), at time (*t*), and frequency (λ).

$$\begin{array}{l}p_{t,\lambda } = {\Bbb R}\left( {{\cal F}\left( {x_t} \right)} \right)\cr Noise{\kern 1pt} Power = \left| {p_{t,\lambda } - \left\langle {p_\lambda } \right\rangle } \right|\end{array}$$

### Information theoretic analyses

Information theory is well suited to characterize complex interactions between neural signals as it is model-independent and capable of capturing nonlinear interactions^59^. Information theoretic measures were used to investigate encoding of the click type (i.e. 1^st^ or 2^nd^) by the brain regions alone and in pairs. Mutual information (MI)^60^ between electrode voltage and click type was used to measure encoding of click type from each electrode. Redundancy^59,61^ between the voltages of pairs of electrodes and the click type was used to measure shared encoding of click type by pairs of electrodes.

First, the voltage of each click pair was mean subtracted (250 ms before first click to 350 ms after the second click). Next, clicks that showed large (1 mV) positive or negative deviations in voltage within 250 ms before to 350 ms after the click were removed. Then, the voltage signal was averaged in 10 ms-bins ranging from 250 ms before each click to 350 ms after that click. For each time bin, a histogram of average voltage values was created across trials and click types for each recording. The average voltage values were binned into three adjacent bins (high voltage, medium voltage, and low voltage), the boundaries of which were set by maximizing the mutual information (MI) (see below) between the voltage states and the click type. The click type was discretized into two states: first click and second click.

Each trial represented one of six possible combinations of the three voltage states and the two click type states. The joint probability of each combination of states is noted as *p*(*x*_*i*_, *y*) where *x*_*i*_ is the voltage state of the ith brain region (*X*_i_) and *y* is the state of the click (*Y*). The joint probability of each combination of states was found by dividing the total number of trials that produced a given state combination by the total number of trials. This joint probability distribution was then used to calculate the MI. The MI is given by:

$$MI\left( {X_i,Y} \right) = \mathop {\sum}\limits_{x_i = 1}^3 {\mathop {\sum}\limits_{y = 1}^2 {p\left( {x_i,y} \right)\log _2\left( {\frac{{p\left( {x_i,y} \right)}}{{p\left( {x_i} \right)p\left( y \right)}}} \right)} }$$

where x_i_ refers to the voltage state of the i^th^ brain region and y refers to the click type.

An identical procedure was used to calculate the joint probability distribution for two brain regions and the click state: *p*(*x*_*i*_, *x*_*j*_, *y*), where *x*_*i*_ is the voltage state of the i^th^ brain region ($X_i$),$x_j$ is the voltage state of the j^th^ brain region (*X*_i_) and *y* is the state of the click (*Y*). This distribution was then used to calculate the redundancy. The redundancy is given by:

$$R\left( {X_i,X_j;Y} \right) = \mathop {\sum}\limits_{y = 1}^2 {p\left( y \right)\min _{\left\{ {X_i,X_j} \right\}}\left[ {\mathop {\sum}\limits_{x \in X} {p\left( {x|y} \right)\log _2\left( {\frac{{p\left( {x,y} \right)}}{{p\left( y \right)p\left( x \right)}}} \right)} } \right]}$$

By taking the minimum information for each click state, the redundancy represents the information overlap between both electrode voltage states about the click state.

A two-step null model comparison was used to assess which MI and redundancy results were significant. First, a Monte Carlo approach was used to generate null model data and the information value found in the real data was compared to the distribution of null information values^62^. To generate these null model data, the binned voltage states were randomized across trials following binning via the maximum MI. This process preserved the overall number of each type of state, but disrupted the relationship between the voltage state and the click type state. Using the randomized trials, the joint probability distributions were generated and the information values were calculated as described above. For each information quantity calculated, 10^4^ null model information results were generated. If <100 of these null trials produced information values larger than the value found in the real data (i.e. p < 0.01), the information result was deemed significant and retained. Non-significant results were removed from the analysis. For each animal type and brain region, at least 70% of the information results were significant using this methodology. If only noise were present, we would expect roughly 1% of information results would be significant. This indicates that significant information results were not due to multiple comparisons. Second, information values were calculated for null data generated prior to the voltage binning procedure to ensure that assigning voltage bins using maximum MI did not bias the results. These null data consisted of normally distributed random voltage values which were randomly assigned to a click state (60: click 1, 60: click 2). The entire information analysis was performed on 100 of these models to generate a distribution of chance level information. These chance level distributions are plotted with the information results from the real data for comparison.

Experimenters who were blind to the animal treatment conducted all analyses. All code and data are freely available upon request to PI (Christopher C. Lapish, PhD).

## Results

### MD alters weight when compared to sham animals

MDs weighed more than shams in cohort 1, but in cohort 2, shams weighed more than MDs (Supplementary Figure 1). See Supplementary Materials for detailed results.

### MD impairs RM

Variances between groups were similar based on F tests. No differences in total interaction time between groups were observed (t(21) = 1.081, *p* = 0.2921; Fig. 1b1). There was no interaction overall when examining the time spent with each object between groups (F(1,19) = 0.2913, *p* = 0.5957, Fig. 1b2). However, increases in interaction time with the novel vs. recent object was observed in sham animals (Bonferroni corrected planned-comparison, (t(9) = 3.339, *p* = 0.0043), but not in MD rats (Bonferroni corrected planned-comparison, t(12) = 1.465, *p* = 0.1685; Fig. 2a2).

### MD alters COMT and GAD67 protein expression

Representative western blots from all three brain regions for COMT and GAD67 are shown in Fig. 1c1, 1c2, respectively. When assessing normalized total COMT, COMT was significantly decreased in MDs compared to shams in the mPFC (t(12) = 6.231, *p* < 0.0001) and TC (t(12) = 2.930, *p* < 0.0126), but not in the vST (t(12) = 1.346, *p* > 0.2); Fig. 1d1). Normalized total GAD67 was significantly decreased in MDs compared to shams only in the TC (t(11) = 2.859, *p* = 0.015) (Fig. 1d2).

### General properties of electrophysiological recordings

Placement of probes is shown in Supplementary Figure 2. From visual inspection of the voltage traces, it appeared that MDs had increased variability of the dEEG signal compared to shams (Fig. 2b). Fig. 2b1 is a randomly selected raw voltage trace is and the change in voltage between adjacent time points in 2b1 is shown in 2b2. Fig. 2c shows the mean power spectra from the mPFC, vertex, and TC between groups. Fig. 2d1, 2d2 shows the mean spectrograms from the mPFC for each group. The first click occurred at 0.6 s and the second at 1.1 s. These findings are similar across brain regions (vertex and TC; data not shown). Noise power is quantified in Fig. 2e, where an increase was observed in MDs over a number of frequency bands compared to shams.

**Fig. 3:**
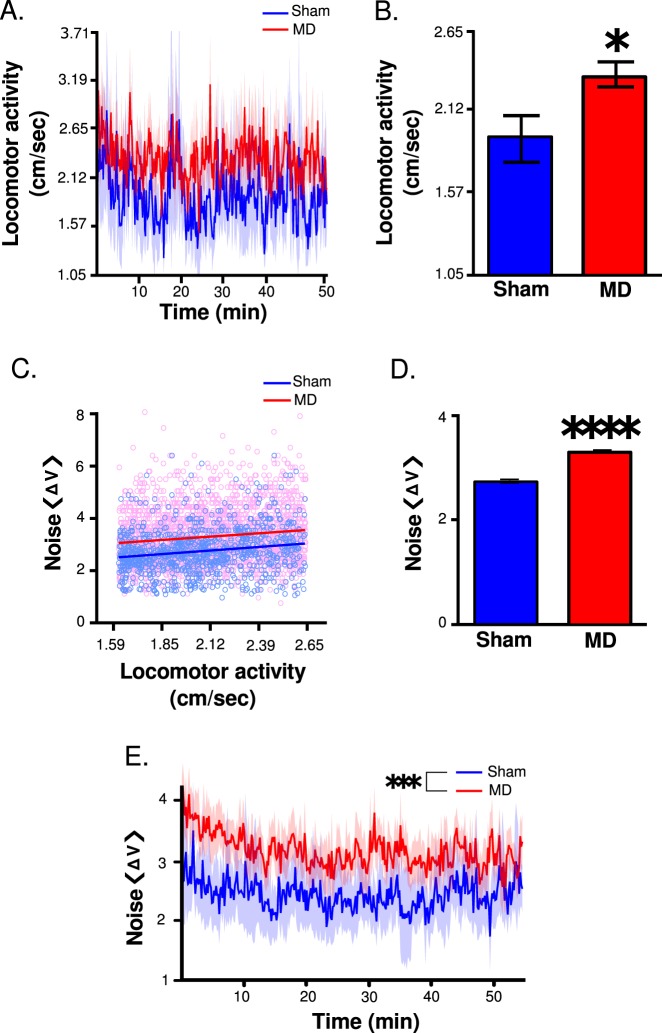
Locomotor activity and differences in bioelectric noise following MD or sham. **a** Mean locomotor activity (solid line) during electrophysiological recordings ± SEM (shaded portion), **b** with significantly increased activity in the MD group (Unpaired two-tailed *t*-test, *p < 0.05; significantly different than sham). **c** Correlation between $\left\langle {\Delta V} \right\rangle$ from the TC and locomotor activity when locomotor activity was held constant in MD and sham groups. **d** An increase in $\left\langle {\Delta V} \right\rangle$ is observed when locomotor speed is held constant. (Unpaired two-tailed *t*-test, ****p < 0.0001, significantly different than sham group). All data are depicted as mean ± SEM. **e** Bioelectric noise (solid line) ± standard deviation (SD) (shaded portion) in the TC throughout the recording session

Locomotor activity was increased at the beginning of the recording in both groups (Fig. 3a). However, when collapsed over time during the entire recording session, MDs exhibited increased locomotor activity relative to shams (main effect of treatment, F(1,7871) = 332.68, *p* < 0.00001, Fig. 3b). These data indicate that MD induced hyperlocomotion in rats, which may model the psychomotor agitation phenotype observed in SZ.

A correlation between $\left\langle {\Delta V} \right\rangle$ signal in the TC and locomotor activity was observed in both groups (*R*^2^ = 0.0431, p < 0.0001, Fig. 3c). However, when locomotor activity was included as a covariate to assess the effects of MD on the TC $\left\langle {\Delta V} \right\rangle$, a treatment X locomotor activity interaction was observed (F(1,7795) = 79.61, *p* < 0.0001), which prevented the direct comparison of each treatment group. The analysis was then restricted to times where each animal was moving between 1.6 and 2.6 cm/s, thus eliminating the aforementioned treatment X locomotor interaction (F(1,3556) = 0.07, *p* = 0.7932) and revealing a main effect of treatment (F(1,3556) = 221.91, *p* < 0.00001; Fig. 3c). Thus, when movement was held constant across each treatment group an increase in the $\left\langle {\Delta V} \right\rangle$ portion of the signal was still observed in MDs, indicating that differences in $\left\langle {\Delta V} \right\rangle$ signal were not exclusively attributable to changes in locomotor activity (Fig. 3d). When movement was held constant, identical results were observed across the other two recording sites (data not shown) (mPFC, main effect of treatment F(1,3556) = 238.39, *p* < 0.00001), (vertex, main effect of treatment F(1,3555) = 278.24, *p* < 0.00001), indicating that increases in the $\left\langle {\Delta V} \right\rangle$ portion of the electrophysiological recordings in MDs was a cortex-wide, and possibly brain-wide, phenomenon.

When the $\left\langle {\Delta V} \right\rangle$ of the signal was quantified, an increase in MDs relative to shams was observed for all three recording sites demonstrated by main effects of treatment (mPFC, F(1,7871) = 769.01, *p* < 0.00001, data not shown; vertex, F(1,7871) = 768.49, *p* < 0.00001, data not shown; TC, F(1,7871) = 801.73, *p* < 0.00001; Fig. 3e). While no effects of time were detected in $\left\langle {\Delta V} \right\rangle$ for each recording site (F’s(1,7871) < 0.75, *p*’s > 0.05), there was a modest increase in the $\left\langle {\Delta V} \right\rangle$ signal in the initial part of the recording.

### Impaired gating of auditory-evoked potentials in the TC of MD animals

**Fig. 4:**
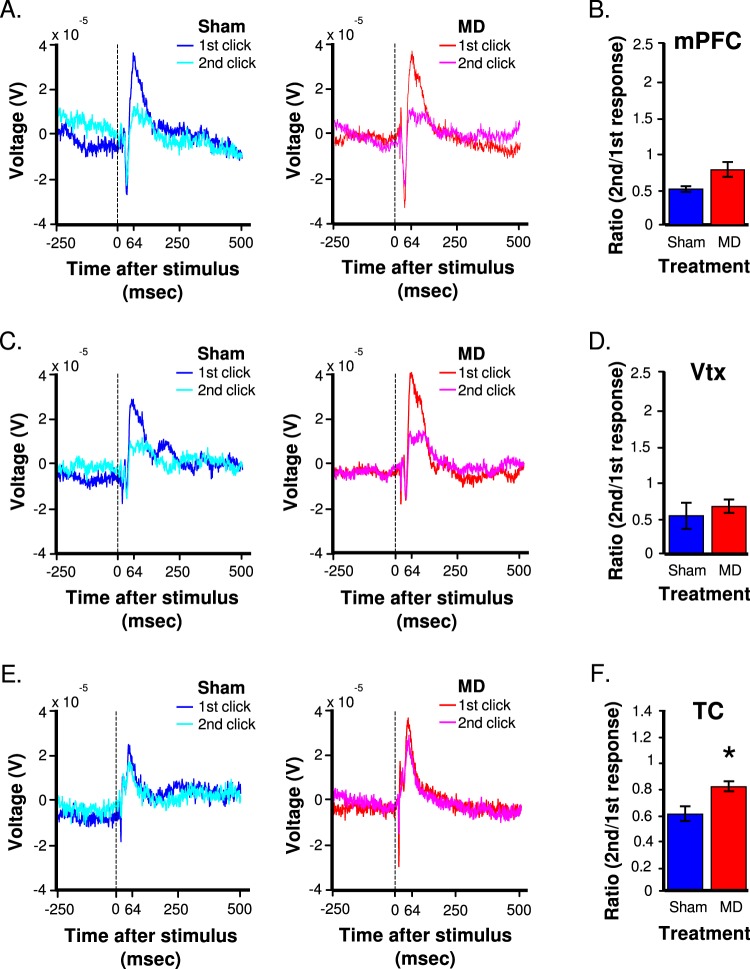
Evoked auditory responses in each brain region during the paired-click paradigm between groups. Mean voltage response to the 1st and 2nd click in sham (blue) and MD (red) animals in the **a** mPFC, **c** vertex, and **e** TC. Vertical dashed lines represent onset of the click. Gating ratio in **b** mPFC, **d** vertex, and **f** TC between groups. (Unpaired two-tailed *t*-test, *p < 0.01, significantly different than shams). All data are depicted as mean ± SEM

Auditory-evoked responses in each brain region were characterized by a tri-phasic response, which consisted of a ~14 ms and ~34 ms latency negative going voltage deflections, followed by a ~64 ms latency positive going deflection. When collapsed across treatment groups, heterogeneity in the magnitude of the peak of the three components of the voltage response across brain regions was observed (brain region×peak interaction, F(4431) = 19.93, *p* < 0.001; Fig. 4a, 4c, 4e). The main difference amongst the evoked responses was the change in the amplitude of the second negative going peak, which was different among each brain region with the mPFC having the largest voltage deflection, followed by the vertex and TC (Tukey’s post-hoc, *p*’s < 0.014).

A brain region×click interaction was observed (F(2,431) = 3.18, *p* < 0.05) indicating a difference in auditory gating across the three brain regions when collapsed across groups. Multiple comparisons revealed differences in the first vs. second click in the mPFC and vertex (Tukey’s post-hoc, p’s < 0.0075) but no differences in the TC (Tukey’s post-hoc, *p* > 0.05), indicating weaker auditory gating at this site.

To determine the effects of MD on auditory gating, the gating ratio was assessed (Fig. 4b, 4d, 4f). A main effect of treatment was observed across all brain regions (F(1,71) = 4.52, *p* = 0.0373), which indicated that the gating response was blunted overall in MD animals. Bonferroni corrected pairwise planned comparisons indicated that there was a significant difference in gating response between groups in the TC only (independent samples two-tailed *t*-test, t(22) = −2.6898, *p* = 0.01338) (Fig. 4f). Thus, the weaker gating ratio observed when collapsed by treatment was driven by a decreased gating ratio in the TC of MD animals. However, since weak gating was observed in the TC to begin with the practical importance of this effect is questionable.

### Reduced information content and sharing is observed in MDs

**Fig. 5:**
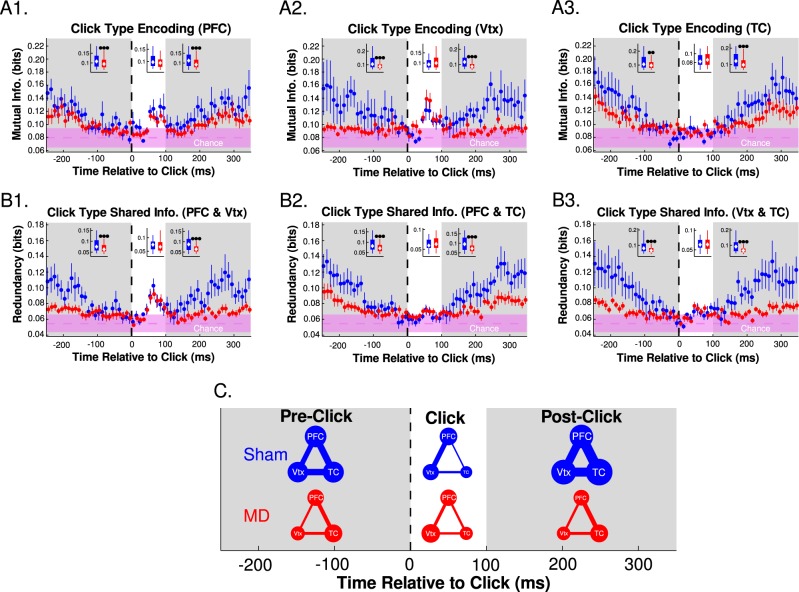
Click type encoding is less stable in MD animals. **a** Click type (1st or 2nd) encoding measured by mutual information between electrode voltage and click type (bin size: 10 ms, dots: mean, error bars: SEM, chance region: median and Standard Deviation (fringe) of null model data). Inset (data collapsed in time regions): gray background (pre-click and post-click); white background (during click), (box plots (5th percentile, interquartile range, median, 95th percentile) Mann–Whitney, *p < 0.05, **p < 0.01, ***p < 0.001). **b** Shared click type encoding between pairs of brain regions as measured by redundancy (inset and chance region: identical structure to (**a**)). **c** Large dots represent more information and thicker lines represent more shared information. Dot radius and line width linearly scaled to median values in (**a**) and (**b**) insets

MI results indicated that the click type could be predicted based on the recorded voltage significantly above chance. Consistent with the auditory gating analysis performed on dEEG voltage changes, both mPFC (Fig. 5a1) and vertex differentiated click type (Fig. 5a2) within 100 ms of click initiation, whereas TC did not (Fig. 5a3). Prior to a click and >100 ms after clicks, shams showed higher click type encoding, suggesting information about the click persisted longer or was more stable in shams compared with MDs.

Redundancy was measured to assess shared encoding between the voltage states of two brain regions and the click type. High redundancy implied the voltage signals from each brain region 1) predicted the click type well individually and 2) predicted the same click type(s) well. Shams showed higher redundant encoding before clicks and more than 100 ms after clicks compared to MDs. Increased shared encoding within 100 ms after a click between mPFC and vertex was apparent (Fig. 5b1), but not in the other pairings (Fig. 5b2, b3). This result was due to the lack of click type encoding shortly after clicks in TC. These results indicate that a stable representation of click type observed in shams is also shared across brain regions.

## Discussion

These data identify a model of early life stress with high translational potential when considered in combination with human studies that observe changes in DA and GABA systems, bioelectric noise, and RM in patients with SZ^40,63^. In the current study, the changes in protein expression and brain function may be a critical underlying factor that influences the flow of information between brain regions, thus leading to abnormalities in integrating sensory information and, ultimately, could cause mild impairment in cognitive function. Collectively these data provide insight into how early life trauma leads to alterations in neural function, which may underlie the increased probability of developing psychopathology later in life.

In the current study, MDs did not spend significantly more time with the novel object compared to the familiar, suggesting that this single 24-h deprivation period produced long-lasting but mild impairments in RM. Similarly, impairments in RM following MD is observed in animals undergoing NOR on PD60 or PD82 following a 4-h retention period^22,64^. These two studies utilized long retention periods, while another study found no impairment after a 3-min retention period^17^. Interestingly, NOR tests that utilize < 5 min retention periods are thought to measure working memory, whereas retention periods of 2-h or 24-h measure short-term and long-term memory, respectively^65^. Therefore, it is possible that MD does not induce impairments in working memory in adulthood, but it does influence short-term or long-term memory.

Deficits in a NOR task that utilized a 4-h retention period were not observed following MD on PD70^23^. However, in these experiments, animals received multiple injections of leptin during early post-natal life, which may have somehow protected them from producing deficits in RM in adulthood. Another study found no differences in RM following a 1-h retention period when testing juvenile animals^21^. Considering that RM develops as the brain matures^66^, it is not surprising that no differences were observed between juvenile control animals and MD animals.

In rodents, RM is impaired following transient inactivation and/or permanent lesions of the HC^67^, perirhinal cortex^68,69^, PFC, and entorhinal cortex^69–72^. Specifically, inactivation of CA1 immediately prior to the familiarization phase of a NOR task impairs RM assessed 24 h later thus highlighting the critical role of the HC in NOR^73^. In addition, damage to the perirhinal cortex has been shown to decrease RM^74^. Neural activity associated with the encoding of an object, and the location where it existed, has been observed in the dorsolateral PFC of monkeys also outlining a potentially critical role of this structure in RM^75,76^. These data are also supported by the findings that each of these brain regions are part of a larger neural circuit that collectively supports RM^71^. Therefore impairments in RM, as observed following MD, suggests pathological function of these brain regions that persists into adulthood and thus a risk factor for psychosis-related disorders^77^.

Reductions in COMT expression of MD animals were not observed in the vST but were observed in mPFC and TC, which suggests an increase of bioavailable DA in these regions. Previous work has indicated that increased DA transmission reduces NOR performance^78^. Furthermore, individuals homozygous for the Val^158^ allele exhibit deficits in RM and altered patterns of functional activation in the HC and PFC^40^. Therefore, the influence of MD on DA metabolism and how this influences RM warrants further study.

Impairments in DA signaling are observed in SZ, which may be related to variations in COMT expression^79,80^, but see^39^. Locomotor hyperactivity is hypothesized to model the positive symptoms of SZ^81^ and is often associated with increased DA activity within the mesolimbic system^82^. In the current study, MD animals exhibited elevated locomotor activity compared to shams, which is similar to another study showing increased locomotion during adulthood in males that underwent MD^83^. Collectively, these data suggest that alterations in COMT expression may be an important consequence of early life trauma that possibly leads to a variety of impairments, including hyperlocomotion and cognition^40^.

Alterations in a biomarker of GABA function were observed in the TC following MD, which parallels human studies that find broad decreases in GAD67 in SZ^84^. Preclinical models of early life trauma also show decreased GAD67 mRNA and protein expression in the stria terminalis^85^, striatum and PFC^86^. Furthermore, an examination of postmortem tissue from patients found that GABAergic neurons have lower GAD67 expression in the TC^87^, PFC^88^, and anterior cingulate cortex^89^, which suggest a broader pathology than the one observed herein. The reduction of GAD67 in TC suggests that alterations in inhibitory drive may be important for the disturbances in sensory processing following MD. It is possible that changes in GAD67 may also affect NOR since the GABA system is involved in RM^90^.

The paired-click paradigm is used to assess the ability to filter out redundant auditory stimuli, which is typically disrupted in SZ^91^ and is attributed to inhibitory processes that blunt the neural response to the second stimuli^92^. Sensory gating is disrupted by increased activation of the mesolimbic DA system and is also influenced by the GABAergic system^93^. In the current study, sensory gating was weak in the TC in general. However a decrease in the gating ratio was observed in MD animals, which is consistent with previous studies reporting altered auditory processing using MD^28^. In the current study, decreased GAD67 was only observed in the TC of MD animals, suggesting that altered GABAergic signaling might contribute to blunted sensory gating^94^. In addition, MDs had increased levels of bioelectric noise and Noise Power. Similarly, patients exhibit increases in this component of the EEG in PFC^95^, which suggests that they may have more variable neuronal activity and insufficiently precise communication across brain networks^95^.

Auditory gating is a systems level phenomenon that relies on neural processing across brain regions^96^, including the PFC, HC, and auditory cortex^97–99^. In the current study, the voltage gating response was not as robust in the TC compared to mPFC or vertex. No information about click type was detected in the TC, whereas this was detectable in the mPFC and vertex due to the clear presence of auditory gating in these regions. This is consistent with a role of the TC in processing information primarily related to the sensory properties of the stimuli whereas the more rostral cortical regions are likely more important in encoding contextual information about the stimuli. In shams, increases in click type encoding were observed prior to and after each click, suggesting that, perhaps, contextual information about the click was encoded during these times. Intriguingly, the vertex recordings in MDs exhibited no information about click type in the absence of the click, suggesting that MD induced persistent alterations in auditory gating, thereby producing changes in these neural circuits that prevented the maintenance of click type information.

A similar phenomenon was observed in the information redundancy analysis. Shared information was reduced following MD and was most pronounced between the mPFC-vertex and TC-vertex. These data might shed light on the underlying changes in neural processing that lead to a mild impairment in RM in MD. Impairments in the ability of each brain region to maintain and share information in the absence of stimuli could reflect alterations in neural processing that lead to less robust representation of each object, preventing the detection of the novel one. Importantly, these data suggest that the deficits in auditory gating are not attributable to deficits in early sensory processing but rather a systems level phenomenon that is required to map contextual information to sensory stimuli.

As mentioned previously, human and animal studies support that altered neurodevelopmental processes can be manifested as psychopathology later in life^100,101^. There is recent evidence supporting that inflammation can play a role in altering early brain development. In animals, maternal immune activation using Poly (I:C), a synthetic analog of double-stranded RNA that mimics the response of an acute viral infection^102^, has been shown to increase inflammatory cytokines and alters microglia and evokes a phenotype that models certain aspects of SZ in adulthood^103–106^. The fact that these changes emerge in adulthood could be attributable to this structure not fully developing until this time^107^. Although not measured in the current study, MD could have caused changes in behavior, protein expression, and physiology due to the early effects of neuroinflammation caused by stress.

As a future direction, assessing the rat brain acutely and in adulthood following MD would allow for the immediate and long-term consequences of this procedure to be disambiguated. One study, however, has evaluated the short-term and long-term consequences of MD and found that following an identical 24-h MD period, hippocampal BDNF mRNA was downregulated, and BDNF protein levels and NMDA receptor subunit expression were decreased only in adulthood but not 2, 6, or 24 h following MD^11^. Since BDNF expression, which is regulated by glutamate, and NMDA receptors are both important modulators of synaptic plasticity^108^, these data by Roceri et al.^11^ indicate that an early life traumatic event induces impairments in brain function that are long lasting possibly through these molecular changes.

Taken together these data identify changes in neurobiology and brain function that are a consequence of early life trauma. Each of the phenotypic alterations reported herein provide important clues as to how early life trauma increases the risk for a neuropsychiatric disorder and represent a possible target for intervention. The fact that MD evoked changes in protein expression along with neural activity suggests these measures may be driven by common epigenetic factors that can be influenced following early life trauma. Furthermore, when considering the similarities in the phenotype evoked by MD and SZ^28^, these data identify a set of measurements that could be further explored as an endophenotype of this condition.

## Electronic supplementary material

Supplemental Material(DOCX 14 kb)

Supplemental Figure 1(PDF 18 kb)

Supplemental Figure 2(PDF 665 kb)
