# Correction: Maternal deprivation induces alterations in cognitive and cortical function in adulthood

**DOI:** 10.1038/s41398-018-0207-6

**Published:** 2018-07-31

**Authors:** Sarine S. Janetsian-Fritz, Nicholas M. Timme, Maureen M. Timm, Aqilah M. McCane, Anthony J. Baucum, Brian F. O’Donnell, Christopher C. Lapish

**Affiliations:** 1 Department of Psychology, Indiana University-Purdue University Indianapolis, Indianapolis, IN USA; 2 Department of Biology, Indiana University-Purdue University Indianapolis, Indianapolis, IN USA; 3 Department of Psychological and Brain Sciences, Indiana University, Bloomington, IN USA; 4 Indiana University School of Medicine Stark Neuroscience Institute, Indianapolis, IN USA; 5 Indiana University-Purdue University Indianapolis School of Science Institute for Mathematical Modeling and Computational Sciences, Indianapolis, IN USA

Correction to: *Translational Psychiatry* ; 10.1038/s41398-018-0119-5; published online 27 Month 2018.

The original version of this Article omitted the author Maureen M. Timm from the Department of Psychology, Indiana University-Purdue University Indianapolis, Indianapolis, IN, USA.

Furthermore affiliation 1 was inadvertently repeated in the affiliation list. These errors have now been corrected in the HTML and PDF versions of the Article.

