# Supplementary material for: Maternal deprivation induces alterations in cognitive and cortical function in adulthood
Source: Transl Psychiatry. 2018 Mar 27;8:71. doi: 10.1038/s41398-018-0119-5 (PMC5913289; doi:10.1038/s41398-018-0119-5)
Supplement: Supplementary file 1 — Supplemental Material(DOCX 14 kb) [file 41398_2018_119_MOESM1_ESM.docx]

**Supplementary Materials**

**Maternal Deprivation Induces Alterations in Cognitive and Cortical Function in Adulthood**

**Running Title: Developmental perturbation of cortical function**

*Sarine S. Janetsian-Fritz, Nicholas M. Timme, Maureen M. Timm, Aqilah M. McCane, Anthony J. Baucum II, Brian F. O’Donnell, Christopher C. Lapish

**Results**

***MD alters weight when compared to sham animals***

MD rats weighed more than shams in cohort 1 (treatment X day interaction (F(7,147)=6.83, p<0.0001)) (Supplementary Figure 1, left shaded region). However, in cohort 2, sham rats weighed more than MD rats (treatment X day interaction (F(42,567)=3.92, p<0.0001)) (Supplementary Figure 1, right shaded region). However, family accounted for a larger percent of the variance (66.7%) compared to the treatment (18.9%). Therefore, the effects observed herein of MD on weight are small compared with familial effects.

**Figure Legends**

**Supplementary Figure 1*:*** Weight data (in grams) taken from postnatal day (PD) 29-71 in cohort 1 and from PD75-85 in cohort 2. All data are depicted as mean ± SEM.

**Supplementary Figure 2:** Coronal sections of the (A) mPFC (from +4.20 mm to +2.20 mm), (B) vertex (from -2.56 mm to -4.52 mm), and (C) TC (from -3.30 mm to -5.60 mm) to depict the unilateral placement of probes (mPFC: AP, +3.2; ML, +0.6; DV, 0.0; vertex: AP, -4.0; ML, +1.0; DV, 0.0; TC: AP, -4.5; ML, 0.0; DV, -4.0, relative to bregma). Open circles represent placements for sham animals (*n=12*) and closed circles represent placements for MD animals (*n=24*).
