# Supplementary figures and images for: Maternal deprivation induces alterations in cognitive and cortical function in adulthood
Source: Transl Psychiatry. 2018 Mar 27;8:71. doi: 10.1038/s41398-018-0119-5 (PMC5913289; doi:10.1038/s41398-018-0119-5)

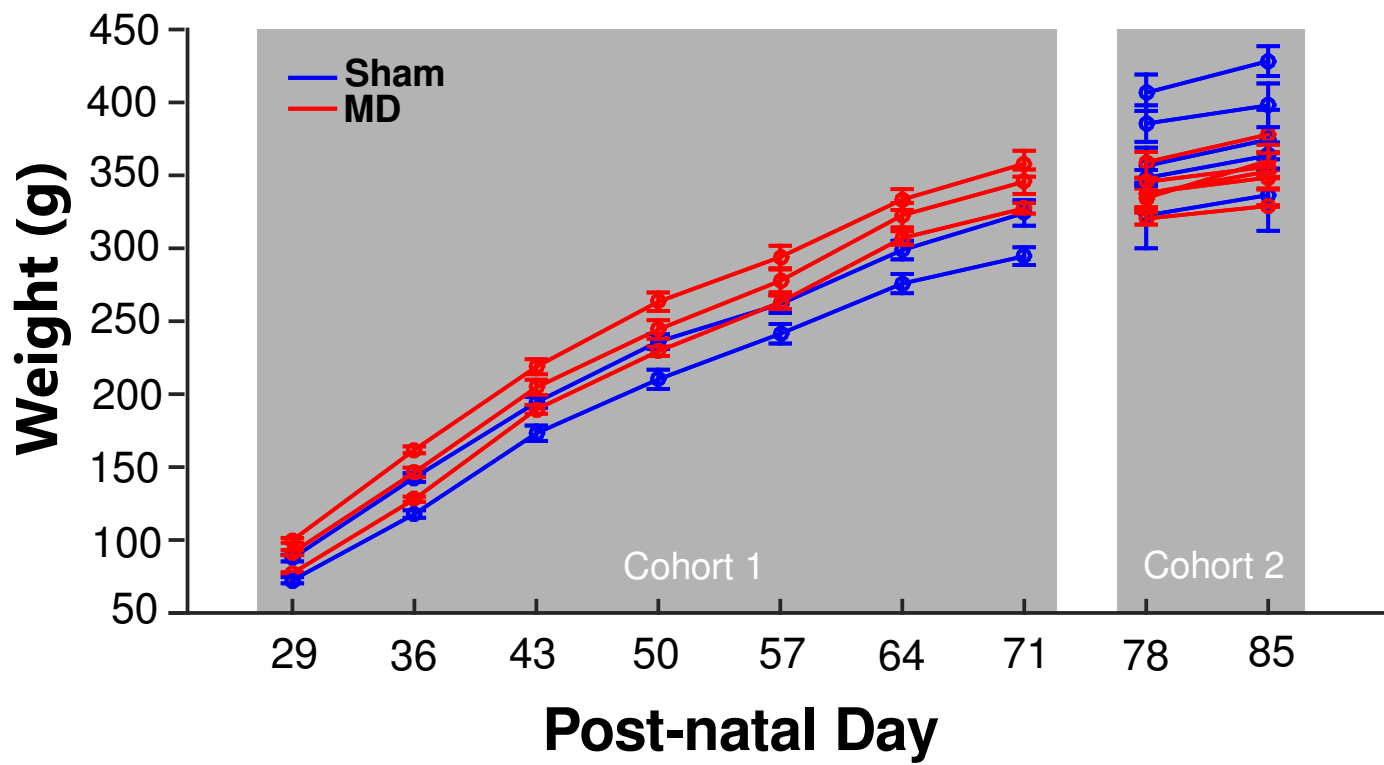

Supplement: Supplementary file 2 — Supplemental Figure 1(PDF 18 kb) [file 41398_2018_119_MOESM2_ESM.pdf]

A.

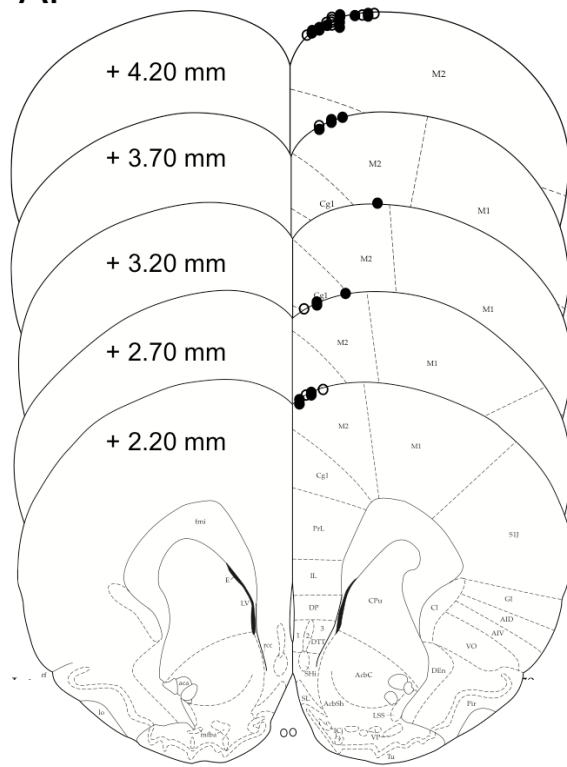

PFC

B.

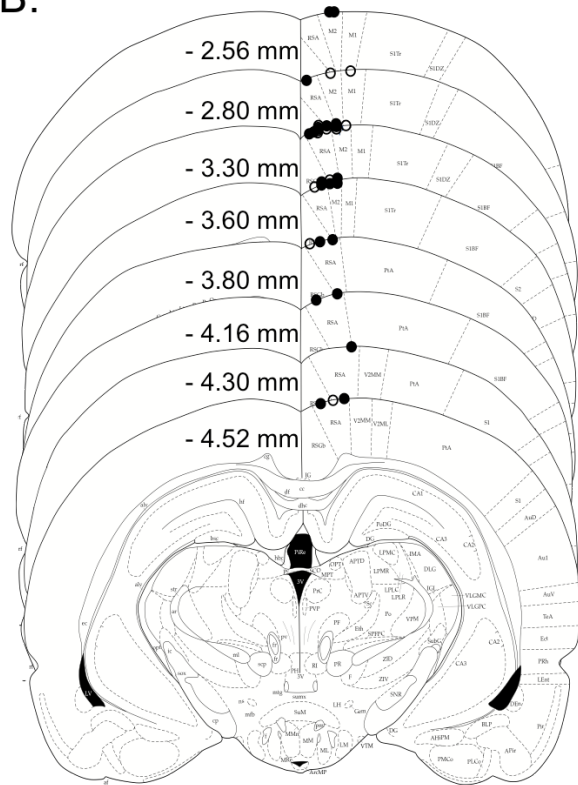

Vertex

C.

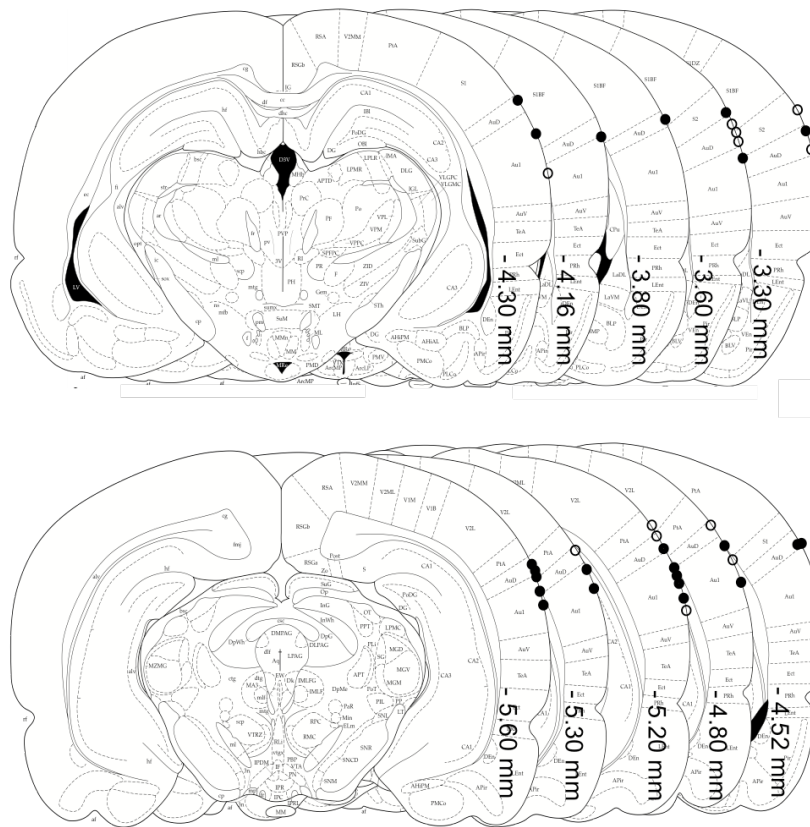

TC

Supplement: Supplementary file 3 — Supplemental Figure 2(PDF 665 kb) [file 41398_2018_119_MOESM3_ESM.pdf]
